# Successful integration of MRI derived scar distribution during VT ablation procedures. Initial experience in 12 patients

**DOI:** 10.1186/1532-429X-11-S1-P34

**Published:** 2009-01-28

**Authors:** Rob J van der Geest, Adrianus P Wijnmaalen, Lucia JM Kroft, Hans-Marc J Siebelink, Jeroen J Bax, Albert de Roos, Martin J Schalij, Johan HC Reiber, Katja Zeppenfeld

**Affiliations:** Leiden University Medical Center, Leiden, Netherlands

**Keywords:** Ventricular Tachycardia, Right Coronary Artery, Registration Error, Left Main, Late Enhancement

## Introduction

Substrate based ventricular tachycardia (VT) ablation requires extensive electroanatomical voltage mapping (EAM) to delineate the myocardial scar and its border zone. Integration of three-dimensional (3D) information from late enhancement (LE) MRI may be feasible and may provide additional information on VT substrate mapping. This study was conducted to test the feasibility of merging 3D MR derived scar information during the VT ablation procedure.

## Methods

In 12 patients (all men; age 64 ± 10) referred for catheter ablation of VT late after myocardial infarction CMR was performed 2–3 days prior to VT ablation. LE MR was performed in multiple slices covering the complete LV in short-axis (slice thickness 10 mm, 5 mm overlap) and long-axis views (two-chamber, four-chamber views, slice thickness 12 mm, 6 mm overlap). In addition, a black-blood imaging sequence was used to image the proximal aorta and the origin of the left main (LM) and right coronary artery (RCA). In the short-axis LE scan, endocardial and epicardial boundaries were derived semi-automatically. The myocardium was divided in an endocardial and epicardial layer and for each layer, at each location, the extent of scar was computed. Subsequently, 3D endocardial and epicardial surface meshes were constructed. Mesh points were color coded based on the extent of scar at the respective myocardial location and layer. In addition, 3D meshes were constructed of the ascending aorta and origin of the LM and RCA.

Prior to VT ablation the generated meshes were imported into the 3D mapping system (CARTO, research version). Based on the 3D catheter tracking capabilities of this system, the MRI derived meshes were registered with the endocardial EAM using the LM position and the endocardial surface as landmarks. After the procedure, 3D mapping point positions and the corresponding bipolar electrogram voltages were saved and superimposed on the original LE MRI data for comparison. Presence of scar from EAM was based on a cut off value of 1.5 mV.

## Results

**Figure 1:**
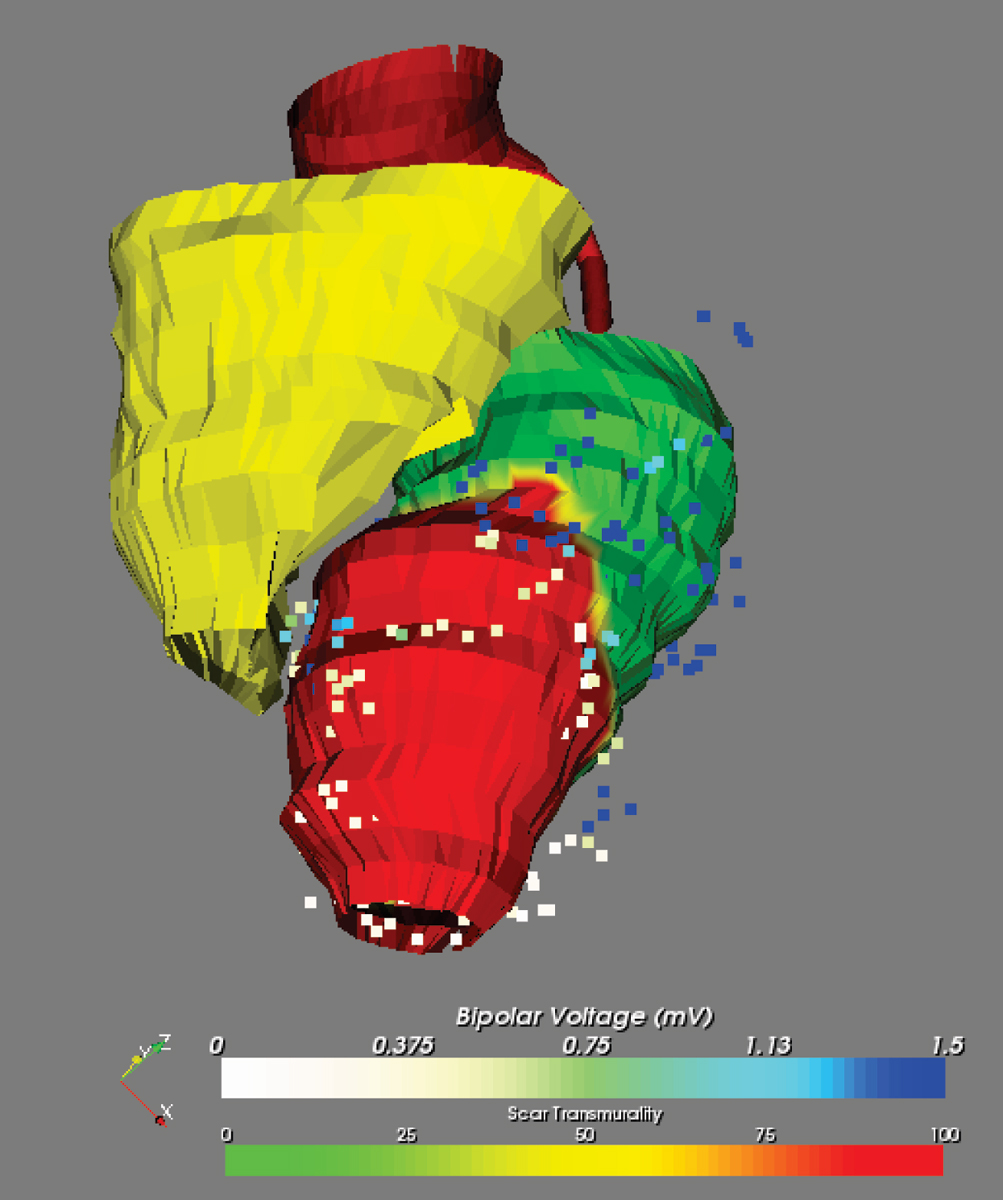
**MRI derived surface mesh depicting the LV endocardial (color coded), RV endocardial (in yellow), aorta (in red) and RCA (in red) anatomy**. Coloring of the LV endocardial surface is used to indicate the transmural extent of scar in the endocardial layer of the myocardium (green = 0%, red = 100%). The square dots indicate the mapping locations obtained during the VT ablation procedure. Color coding is used to indicate the bipolar electrogram amplitude (white ≤ 0 mV, dark blue ≥ 1.5 mV).

**Figure 2:**
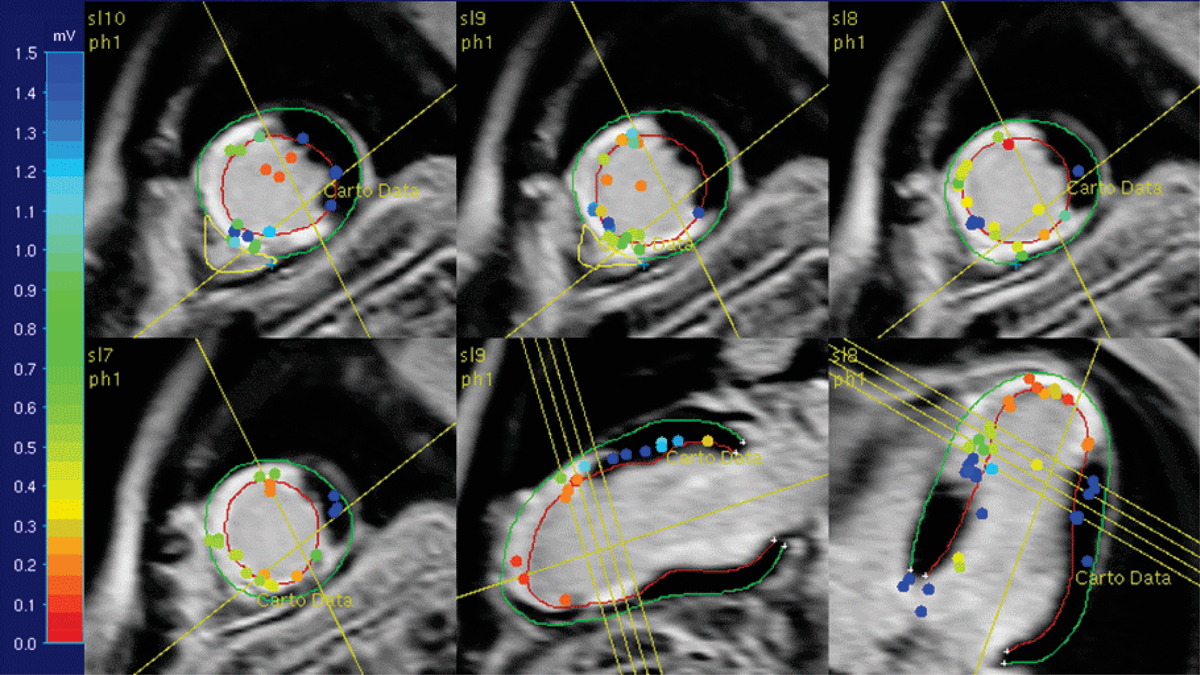
**Late enhancement MRI of the same patient showing transmural scar in the septal-apical region with results of EAM superimposed**. Bipolar voltages are low (<1.5 mV) in the scar region and normal (>1.5 mV) in the healthy myocardium.

Average LV EF was 43 ± 10%; average MRI derived scar size was 11 ± 11% of the LV myocardium. Integration of MRI derived scar information with EAM was successful in all cases. The average number of endocardial mapping points was 264 ± 37. The mean registration error between the MRI derived endocardial surface and EAM was 4.4 ± 3.0 mm. In all patients, regions with transmural scar as seen on MRI, were also identified by EAM (bipolar electrogram amplitude <1.5 mV). In the majority of regions without scar, bipolar voltages were >1.5 mV. However, in regions with non-transmural scar as derived by MRI, EAM derived bipolar voltages were either <1.5 mV or >1.5 mV. Figures 1 and 2.

## Conclusion

It was demonstrated that integration of MRI derived scar information into a 3D mapping system is feasible. The observed registration errors are small compared to the expected amount of cardiac and respiratory motion and the slice thickness of the LE MRI acquisition. The additional information provided by MRI, especially in regions of non-transmural scar, may be helpful to assess the anatomical substrate of VT and to improve the efficiency and outcome of VT ablation procedures.

